# Murine studies and expressional analyses of human cardiac pericytes reveal novel trajectories of SARS-CoV-2 Spike protein-induced microvascular damage

**DOI:** 10.1038/s41392-023-01489-2

**Published:** 2023-06-02

**Authors:** Elisa Avolio, Prashant K. Srivastava, Jiahui Ji, Michele Carrabba, Christopher T. W. Tsang, Yue Gu, Anita C. Thomas, Kapil Gupta, Imre Berger, Costanza Emanueli, Paolo Madeddu

**Affiliations:** 1 Bristol Medical School, Translational Health Sciences, University of Bristol, Bristol, UK; 2 National Heart & Lung Institute, Imperial College, London, UK; 3 School of Biochemistry, University of Bristol, Bristol, UK

**Keywords:** Gene expression analysis, Translational research, Infectious diseases, Cardiovascular diseases, Preclinical research

**Dear Editor**,

The Coronavirus disease 2019 (COVID-19) pandemic has caused over 670 million cases and 6.7 million deaths worldwide, many of which are attributed to cardiovascular complications. Virus-induced endothelial damage, endothelial barrier dysfunction, thrombosis, and cytokine storm are implicated in heart and multi-organ failure. The prognosis is worsened by comorbidities, including diabetes and arterial hypertension, characterized by an inflammatory and pro-thrombotic milieu and upregulation of total and glycosylated Angiotensin-Converting Enzyme 2 (ACE2) in cardiomyocytes.^1,2^

Cardiac pericytes represent a preferential target of SARS-CoV-2 infection.^3,4^ These perivascular cells preserve vascular integrity through physical and paracrine crosstalk with capillary endothelial cells. Pericyte dysfunction and detachment favor the SARS-CoV-2 to spread from the bloodstream and damage the myocardium.^5^

SARS-CoV-2 infection starts with the engagement of the Spike (S)-protein with its cellular ACE-2 and CD147 receptors. Due to the homology with human proteins, the S-protein also acts as a natural ligand activating the ERK1/2 MAPK signaling pathway in cardiac pericytes.^6,7^ Some evidence suggests that the S-protein, CD147, cyclophilin, and MAPK axis are essential in triggering the cytokine storm.^7^ However, an in vivo demonstration of the S-protein’s direct damaging effect on cardiac pericytes is lacking.

The present study investigated the acute effects of intravenously injected S-protein on the heart microvasculature of otherwise healthy mice. Moreover, we analyzed the expressional changes caused by the S-protein in primary cultures of human cardiac pericytes using bulk RNA-Sequencing. Finally, the RNA-Sequencing data were cross-referenced with single-nuclei (sn)-RNA-Sequencing datasets of COVID-19 patients’ hearts to determine how expressional changes after SARS-CoV-2 infection overlap with those caused by the S-protein alone.

Nine-week-old healthy CD1 mice (6 male, 6 female) were randomized to receive either 10 µg endotoxin-free S-protein resuspended in 100 µL sterile PBS or PBS only, intravenously. They were culled three days later for molecular and histological analyses (Fig. 1a). S-protein immunoreactive levels in the circulation were like those reported in COVID-19 patients early after infection and before seroconversion (26.8 ± 7.9 ng/mL).^7^ Immunohistochemistry of the hearts demonstrated that the S-protein, although not altering the capillary density, increased the fraction that expresses ICAM-1, an adhesion molecule implicated in leucocyte-endothelial interactions (Fig. 1b) and remarkably reduced the pericyte density, coverage, and viability (Fig. 1c–e). SARS-CoV-2 can trigger direct or indirect activation of all three complement pathways.^8^ Here, we show that the in vivo administration of S-protein increased complement-activated C5a protein levels in peripheral blood and the heart (Fig. 1f, g). Moreover, the S-protein increased the heart’s abundance of CD45+ immune cells (23.9 ± 2.2 cells/mm^2^ vs. 9.2 ± 1.9 cell/mm^2^ in PBS-treated mice), specifically Ly6G/6C+ neutrophils/monocytes (Fig. 1h) and F4/80+ macrophages (Fig. 1i). Leucocytes can crawl along pericyte processes to enlarged gaps between adjacent pericytes in an ICAM-1-dependent manner during inflammation. Controls for immunohistochemistry stainings are provided in Supplementary Fig. S1.

**Fig. 1:**
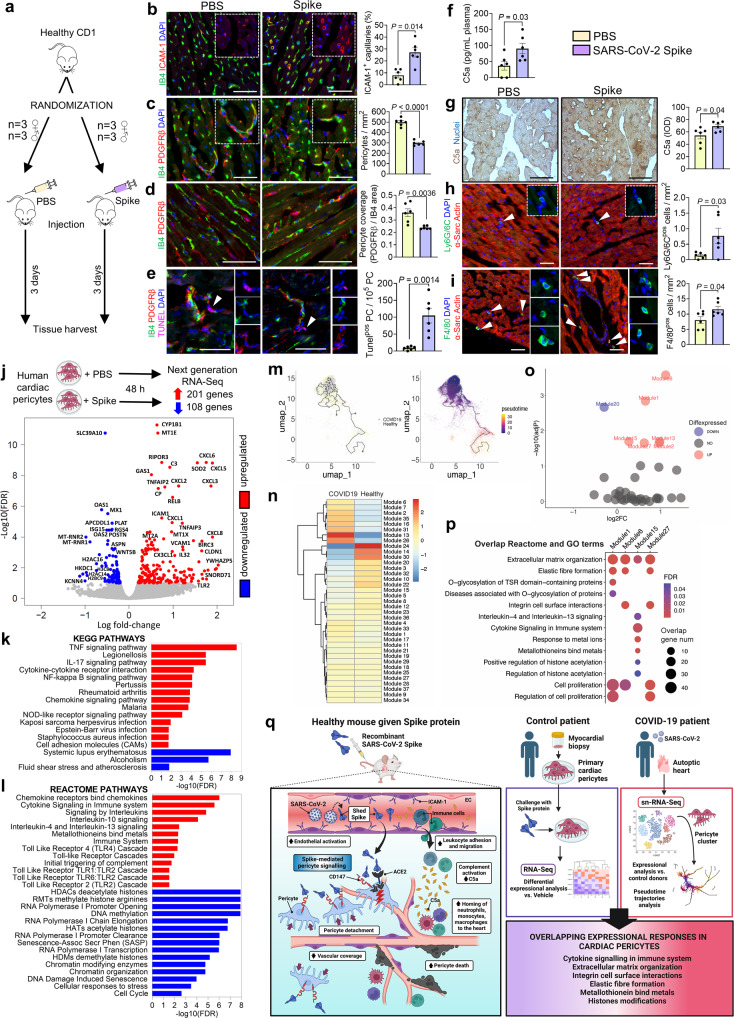
**a**–**i** Injection of S-protein in vivo in mice. **a** Experimental design of the in vivo study in mice. **b** Representative immunofluorescence images of mice hearts showing capillaries (IB4, green) and activated endothelium (ICAM-1, red). Bar graphs summarize the quantitative analysis of capillaries positive for ICAM-1, expressed as a percentage of total vessels. **c** Representative immunofluorescence images showing capillaries (IB4, green) and pericytes (PDGFRβ, red). Bar graphs summarize the quantitative analysis of pericyte density. **d** Representative immunofluorescence images showing longitudinal capillaries (IB4, green) covered by pericytes (PDGFRβ, red). Bar graphs report the quantitative analysis of pericyte coverage. **e** Representative immunofluorescence images of mice hearts showing endothelial cells (IB4, green), pericytes (PDGFRβ, red), and TUNEL-positive nuclei (apoptotic nuclei, magenta). Bar graphs report the quantification of TUNEL+ pericytes. **f** Measurement of C5a in mice plasma using ELISA. **g** Immunohistochemistry/DAB staining and a bar graph showing the accumulation of the activated complement factor C5a in the mice hearts. Nuclei are shown in blue (Haematoxylin). The graph reports the integrated optical density (IOD) values. Representative immunofluorescence images of mice hearts showing the presence of neutrophils/monocytes (**h**—Ly6G/6 C, green) and macrophages (**i**—F4/80, green). Cardiomyocytes are labeled with α-Sarcomeric Actin (red). Bar graphs report the density of Ly6G/6 C+ neutrophils/monocytes and F4/80+ macrophages. In all immunofluorescence images, DAPI labels nuclei in blue. For all images, the scale bar is 50 μm. For all analyses, *n* = 6 per group. All data are presented as individual values and means ± SEM. Statistical tests: after a normality test, an unpaired *t*-Test was applied. **j**–**l** RNA-Sequencing analysis of human cardiac pericytes challenged with the S-protein in vitro. *n* = 3 patients. **j** Experimental design and volcano plot showing transcripts differentially expressed in S-protein-treated (5.8 nM) human cardiac pericytes vs. PBS vehicle-treated pericytes. The terms of the most relevant genes were reported. **k** Bar graph indicating all differentially expressed KEGG pathways. **l** Bar graphs indicating the most relevant differentially expressed Reactome pathways. FDR = false discovery rate. Genes were considered differentially expressed for FDR ≤ 0.1. **m**–**p** Sn-RNA-Sequencing analysis of pericytes from COVID-19 patients’ hearts. n = 22 COVID patients, *n* = 25 controls. **m** Plots show the ordering of pericytes in pseudo-time. The starting point of pseudo-time is from the pericytes of healthy donors. **n** A heatmap summarizing the mean expression of normalized unique molecular identifiers (UMIs) of genes in the modules resulting from the pseudo-time analysis. **o** A volcano plot showing fold-change of module expression (COVID-19 compared to healthy donors) and enrichment significance of each module and differentially expressed genes from bulk RNA-Sequencing comparing PBS-vehicle and Spike. **p** A plot summarising overlapped/similar Reactome and Gene Ontology terms overrepresented in each module and differentially expressed genes in bulk RNA-Sequencing. **q** Schematic summarizing major findings and candidate mechanisms underpinning the S-protein damaging action. Left panel: We provide novel evidence that S-protein alone can damage the heart microvasculature of otherwise healthy mice. On one side, the S-protein acts as a ligand activating intracellular pericyte signaling, which results in pericyte detachment, death, and decreased vascular coverage, thus disrupting the coronary microcirculation. On the other, the S-protein triggers endothelial activation (ICAM-1+ endothelial cells), resulting in increased homing of leukocytes to the heart and accumulation of activated complement protein C5a. Right panel: A comparison between the expressional changes induced by the S-protein in primary human cardiac pericytes in vitro and single-nuclei (sn)-RNA-Sequencing pseudo-time trajectories analysis in pericytes extracted from the heart of deceased COVID-19 patients revealed overlapping expressional responses as indicated. These findings suggest that at least some of the in vivo effects of SARS-CoV-2 on human cardiac pericytes may be due to the modulation of inflammatory and epigenetic pathways triggered by the S-protein interaction with its cell surface receptors. The drawing was created with BioRender.com

To further validate the theory of the S-protein acting as a direct transcriptomic influencer, we added it or the PBS vehicle to human primary cardiac pericytes in vitro for 48 h. RNA-Sequencing analysis indicated the differential modulation of 309 RNA transcripts, with 201 genes being up-regulated and 108 genes down-regulated by the S-protein at FDR < 0.1 (Fig. 1j). KEGG pathway analysis showed an overrepresentation of inflammatory pathways, for example, TNF, IL-17, and NF-kappa B signaling pathways, cytokine-cytokine receptor interaction, and cell adhesion molecules (CAMs). Moreover, there was an enrichment for pathways associated with infectious diseases, including Legionellosis, Pertussis, Malaria, Herpes virus, and Epstein-Barr virus infection (Fig. 1k). An overview of the pathway analysis based on the Reactome database further pinpointed the transcriptional induction of cytokine signaling pathways, such as IL-10, IL-4, and IL-13 signaling and Toll-like receptor cascade (Fig. 1l and Supplementary Fig. S2), and the downregulation of pathways implicated in histone deacetylation and methylation and chromatin modification, and RNA polymerase-related mechanisms controlling promoter opening and clearance, transcription, and chain elongation (Fig. 1l and Supplementary Fig. S2). The analysis of modulated biological processes confirmed the upregulation of cellular responses to stress and the downregulation of homeostatic responses associated with healing and angiogenesis processes (Supplementary Fig. S3). A comprehensive list of regulated pathways is provided in Supplementary Dataset 1.

Aiming to dissect clinically relevant targets further, we cross-interrogated the transcriptional landscape of pericytes exposed in vitro to the recombinant S-protein and pericytes from the hearts of COVID-19 patients. Additionally, we employed a pseudo-time inference approach to probe individual genes’ expression dynamics along with the progression of the disease. To this aim, we extracted pericytes from the integrated Seurat, R object (downloaded from Delorey et al., 2021)^9^ using marker genes followed by a pseudo-time analysis of pericytes collected from the heart of COVID-19 patients (Fig. 1m). The pseudo-time analysis allowed the identification of pericyte genes that are differential and co-expressed along the trajectory. This resulted in the recognition of 37 gene clusters (Fig. 1n). Next, to identify common signals between ex vivo and in vivo datasets, we tested for the overrepresentation of expressional changes in pericytes exposed to S-protein and gene clusters in the human heart. We observed that seven gene clusters (1, 2, 6, 13, 15, 20, and 27, FDR < 0.05) significantly overlapped with the expressional changes observed in pericytes exposed to the S-protein experiment (Fig. 1o). Cluster 15 was enriched for cytokine-related pathways, metallothioneins, and regulation of histone acetylation, while clusters 1, 6 and 27 were overrepresented for extracellular matrix organization, elastic fibre formation, and integrin cell surface interactions (Fig. 1p and Supplementary Dataset 2). Studies have reported that COVID-19 can cause cardiovascular complications due to impaired extracellular matrix organisation and reduced elastic fibre levels, potentially leading to blood clots.^10^ These findings suggest a convergence of signals that proteins of the virion envelope mediate at least part of the transcriptional changes induced by the virus in the hearts of infected people. Therefore, some of the in vivo effects of SARS-CoV-2 on human cardiac pericytes may be attributable to the interaction between the S-protein and the host’s transcriptomic program modulating inflammatory and epigenetic pathways.

Finally, we performed drug target enrichment analysis using the LINCS L1000CDS and DrugBank databases. This analysis allowed us to identify drugs that reverse the expressional changes induced by the S-protein in pericytes (Supplementary Dataset 3 and 4). Among the top fifty compounds, we found a prevalence of anti-tumoral, pro-apoptotic, anti-viral, anti-inflammatory and anti-thrombotic drugs, some of which have already been trialed in COVID-19 patients. Although more research is needed to determine if pharmacological interference with the signaling emanating from the S-protein can alleviate COVID-19 outcomes, these data suggest a competitive effect of anti-inflammatory and anti-tumoral drugs. In addition, several compounds like Quercetin or ubiquitin-conjugating enzyme inhibitors may help moderate inflammation by eliminating S-Protein-induced senescent cells.

Findings summarized in Fig. 1q provide novel evidence of the SARS-CoV-2 S-protein’s direct pathogenic action on cardiac pericytes and the heart’s microvasculature. It is plausible that the harmful effects observed in healthy mice three days after a single systemic injection of the S-protein might be intensified in the presence of cardiovascular risk factors and prolonged exposure. These possibilities merit further investigation. Moreover, we showed that the S-protein modifies the transcriptional program of human cells to the virus’ advantage. This new information could have significant implications for the treatment of COVID-19, for instance, using anti-S-protein engineering approaches to protect vascular cells.

## Supplementary information

Sigtrans_Supplementary_Materials.docx

Dataset 1

Dataset 2

Dataset 3 + 4

## Data Availability

The article’s data can be obtained as reasonably required from the corresponding author. The main datasets underlying transcriptomic analyses are provided as supplementary datasets (Dataset 1–4). The bulk RNA-Seq raw data have been deposited in NCBI’s Gene Expression Omnibus and are accessible through GEO Series accession number GSE218644.
