# Supplementary material for: Murine studies and expressional analyses of human cardiac pericytes reveal novel trajectories of SARS-CoV-2 Spike protein-induced microvascular damage
Source: Signal Transduct Target Ther. 2023 Jun 2;8:232. doi: 10.1038/s41392-023-01489-2 (PMC10236384; doi:10.1038/s41392-023-01489-2)
Supplement: Supplementary file 1 — Sigtrans_Supplementary_Materials.docx [file 41392_2023_1489_MOESM1_ESM.docx]

Supplementary Materials for

**Murine studies and expressional analyses of human cardiac pericytes reveal novel trajectories of SARS-CoV-2 Spike protein-induced microvascular damage**

Elisa Avolio, Prashant K Srivastava, Jiahui Ji, Michele Carrabba, Christopher TW Tsang,

Yue Gu, Anita C Thomas, Kapil Gupta, Imre Berger, Costanza Emanueli, Paolo Madeddu

Correspondence to:  [mdprm@bristol.ac.ukx](mailto:xxxxx@xxxx.xxx)

**This PDF file includes:**

Materials and Methods

Figures. S1 to S3

Tables S1 to S2

**Other Supplementary Materials for this manuscript include the following:**

Dataset S1: Supplementary Dataset 1_bulk RNA-seq enrichment_results.xlsx

Dataset S2: Supplementary Dataset 2_snRNAseq modules.xlsx

Dataset S3 and S4: Supplementary Dataset 3+4_drugs.xlsx

Materials and Methods

All chemicals were purchased from Sigma-Aldrich if not otherwise specified.

**Study approval**

*Animal studies:* The animal study was covered by a license from the British Home Office (PPL 1377882) and complied with EU Directive 2010/63/EU. Procedures were carried out according to the principles in the Guide for the Care and Use of Laboratory Animals (The Institute of Laboratory Animal Resources, 1996). Termination was conducted according to humane methods outlined in the Guidance on the Operation of the Animals (Scientific Procedures) Act 1986 Home Office (2014). The report of results is in line with the ARRIVE guidelines.

*Human cell studies:* primary cardiac pericytes were extracted from discarded human myocardial material from surgical repair of congenital heart defects (ethical approval number 15/LO/1064 from the North Somerset and South Bristol Research Ethics Committee. Paediatric patients' custodians gave informed written consent. Donors and sample characteristics are described in **Table S1**. Patients were recruited before the COVID-19 pandemic.

**Administration of SARS-CoV-2 S protein to mice and tissue harvest**

The SARS-CoV-2 S-protein was expressed and purified as described previously^1-3^. Healthy CD1 mice (6 male, 6 female) were purchased from Charles River and used at nine weeks of age. Animals were housed in an enriched environment within a bio-secure unit under a 12 h light/dark cycle, fed with EURodent Diet (5LF5, LabDiet) and given drinking water *ad libitum*. They were randomized to receive either 10 µg endotoxin-free S-protein resuspended in 100 µL sterile PBS, or only PBS as vehicle, *via* a single intravenous (IV) injection in the tail. The dosage and delivery route of the S-protein were determined based on a previous study in mice.^4^ Each experimental group presented equal sex distribution. After 3 days, animals were culled, and tissues were harvested for molecular and histological analyses. Blood was collected with EDTA to separate plasma for analysis of circulating biomarkers. Hearts were stopped in diastole using KCl and perfusion-fixed with PBS-EDTA followed by 4% paraformaldehyde (PFA). After harvest, hearts were fixed in 4% w/v PFA at 4°C overnight, immersed in 30% w/v sucrose in PBS overnight and embedded in OCT (Tissue-Tek® O.C.T. Compound, VWR) for cryopreservation and histological analyses. Five-μm thick sections were cut for immunostainings.

**Measurement of S-protein and C5a in mice plasma**

Blood-EDTA was centrifuged for 15 min at 5,000 *g* at 4 ͦ C for plasma separation. The SARS-CoV-2 (2019-nCoV) Spike ELISA Kit (Sino Biological, Catalog Number: KIT40591) was used according to the manufacturer’s instructions, except for using our own recombinant S-protein (the same that was injected in mice) to generate the standard curve. This allowed an accurate measurement of S-protein in mice plasma. All plasma samples were diluted 1:2 with the kit reagent diluent. For the quantitative measurement of complement C5a in mice plasma, we used the Mouse Complement C5a ELISA Kit (Abcam - ab193718), according to the manufacturer's instructions. All plasma samples were diluted 3-fold.

**Histological analysis of mice hearts**

Cryosections were either post-fixed and permeabilised with ice-cold acetone (VWR) for 5 min at -20 °C, followed by air drying for 30 min and hydration in PBS for 10 min, or underwent hydration and heat-induced antigen retrieval with citrate buffer 0.01 M pH 6, for 15 min at 98 °C, followed by cooling down to 20 °C for 40 min, and rinsing with PBS.

*Fluorescence-based development*. Tissue sections were blocked with 5% v/v normal donkey or goat serum (as appropriate) and incubated with primary antibodies for 16 h at 4 °C. Antibodies are reported in **Table S2**. Secondary antibodies (Alexa 488-, Alexa 568-, Alexa 647-conjugated, produced in goat or donkey) were all purchased from ThermoFisher Scientific and used at a dilution of 1:200 for 1 h at 20 °C in the dark. Slides were mounted using ProLong™ Gold Antifade Mountant with DAPI (ThermoFisher Scientific). For analysis of apoptosis, two TUNEL kits were employed (In situ Direct DNA Fragmentation (TUNEL) Assay Kit (Abcam ab66108) and the ApopTag® Fluorescein In Situ Apoptosis Detection Kit (EMD Millipore S7110)), according to manufacturer’s instructions.

*DAB-based development*. To detect primary rabbit antibodies using 3,3′-Diaminobenzidine (DAB), we used the Rabbit specific HRP/DAB Detection IHC Detection Kit - Micro-polymer from Abcam (ab236469), according to the manufacturer’s instructions. Nuclei were counterstained using Mayer’s Haematoxylin.

Controls omitting primary antibodies and including isotype controls are provided in **Figure S1**.

**Quantification of mice hearts**

Data were acquired using a Zeiss AxioObserver Z1 Microscope equipped with 10 x and 20 x objectives, followed by processing and quantification using ImageJ. To quantify capillary and pericyte densities, 15 random fields were acquired using a total of 200 x magnification from 3 slices. Data were expressed as the number of IB4-positive capillaries or PDGFRβ-positive pericytes per square millimeter of myocardial tissue. Capillaries positive for ICAM-1 were expressed as a percentage of total capillaries. The pericyte coverage was quantified as the ratio between the PDGFRβ and IB4 pixel counts, as previously described.^5^ To quantify apoptosis, 3 to 6 whole slices were analyzed per heart. Data were expressed as the number of TUNEL-positive pericytes per 10^5^ pericyte nuclei. Two whole sections were quantified for CD45, Ly6G/6C and F4/80-positive cells, and results were expressed as the number of cells per mm^2^ of myocardial tissue. The accumulation of C5a in the mice’s hearts was quantified and expressed as integrated optical density (positive signal vs total area).

**Bulk RNA-Sequencing (RNA-Seq) of human cardiac pericytes**

Cardiac pericytes were extracted from human myocardial samples and expanded *in vitro* as previously described.^6,7^ Pericytes from 3 donors were challenged with 1 μg/mL (5.8 nM) S-protein or PBS-vehicle for 48 h in the absence of FBS and growth factors. Total cellular RNA was extracted for next-generation RNA-Seq analysis. Briefly, RNA-sequencing reads were mapped to the Homo sapiens GRCh38 reference genome available on ENSEMBL using the STAR alignment tool (version 2.5.4b). While differential expression analysis was performed using the Bioconductor package EdgeR, which implements a generalized linear model based on a negative binomial test model for RNA-Seq in R.^8^ P values were corrected for multiple testing using BH FDR. A cut-off of FDR ≤0.1 was applied to select differentially expressed genes. KEGG^9^, Reactome^10^ and Biological Process pathways analyses were performed. The RNA-Seq data sets have been deposited in NCBI's Gene Expression Omnibus^11^ and are accessible through GEO Series accession number GSE218644.

**Single nucleus (sn)-RNA-Seq in pericytes extracted from COVID-19 patients’ hearts**

We downloaded sn-RNA-Seq data from Delorey et al., 2021,^12^ where they employed sn-RNA-Seq technologies to characterize cellular states in the hearts of COVID-19 patients.^12^ Briefly, Delorey et al. generated sn-RNA-Seq from COVID-19 infected hearts (n=22) and integrated them with sn-RNA-Seq generated from the donor’s hearts (n=7 from Tucker et al.^13^ and n=18 from Litvinokova et al.^14^) using harmony. We downloaded the integrated sn-RNA-Seq data, extracted pericytes from the integrated object, and performed pseudo-time ordering using Monocle 3 framework.^15-17^

**Functional enrichment of differentially expressed genes**

Functional term enrichment analysis for differentially expressed genes from each comparison (genes from bulk pericytes and sn-RNA-Seq modules) was performed using the overrepresentation analysis module from the R package implementation of WEB-based Gene SeT Analysis Toolkit (WebGestaltR, v0.4.4).^18^

**Drug target enrichment analysis**

Drug target enrichment analysis was performed to identify drugs and small molecules that could reverse the gene signature after exposure to Spike protein. We used L1000CDS^2^, web applications to test drugs that reverse the gene expression signatures.^19,20^ The web servers employ drug target database. The database is a library of integrated network-based cellular signatures (LINCS) L1000, which comprises over a million gene expression profiles of chemically perturbed human cell lines at different drug concentrations.

We also performed non-directional drug target enrichment using DrugBank database implemented in WebGestalt (WEB-based Gene SeT AnaLysis Toolkit) webserver.^18^A drug was considered to be significant if the FDR was < 0.05.

**Statistical analyses**

Continuous variables are presented as means ± standard error of independent samples’ mean (SEM) and individual values. The Kolmogorov-Smirnov normality test was used to check for normal distribution. Continuous variables normally distributed were compared using the unpaired Student's *t*-test (two groups comparison). For data not following normal distribution, we used the unpaired Mann-Whitney test (two groups comparison). Data were analysed using Prism version 9.0. Statistical significance was assumed when P ≤ 0.05.

**References**

1 Avolio, E. *et al.* The SARS-CoV-2 Spike protein disrupts human cardiac pericytes function through CD147 receptor-mediated signalling: a potential non-infective mechanism of COVID-19 microvascular disease. *Clin Sci (Lond)* **135**, 2667-2689 (2021).

2 Toelzer, C. *et al.* Free fatty acid binding pocket in the locked structure of SARS-CoV-2 spike protein. *Science* **370**, 725-730 (2020).

3 Amanat, F. *et al.* A serological assay to detect SARS-CoV-2 seroconversion in humans. *Nature medicine* **26**, 1033-1036 (2020).

4 Nuovo, G. J. *et al.* Endothelial cell damage is the central part of COVID-19 and a mouse model induced by injection of the S1 subunit of the spike protein. *Ann Diagn Pathol* **51**, 151682 (2021).

5 Chintalgattu, V. *et al.* Coronary microvascular pericytes are the cellular target of sunitinib malate-induced cardiotoxicity. *Sci Transl Med* **5**, 187ra169 (2013).

6 Avolio, E. *et al.* Cardiac pericyte reprogramming by MEK inhibition promotes arteriologenesis and angiogenesis of the ischemic heart. *J Clin Invest* **132** (2022).

7 Avolio, E. *et al.* Expansion and characterization of neonatal cardiac pericytes provides a novel cellular option for tissue engineering in congenital heart disease. *J Am Heart Assoc* **4**, e002043 (2015).

8 Robinson, M. D., McCarthy, D. J. & Smyth, G. K. edgeR: a Bioconductor package for differential expression analysis of digital gene expression data. *Bioinformatics* **26**, 139-140 (2010).

9 Kanehisa, M., Sato, Y., Kawashima, M., Furumichi, M. & Tanabe, M. KEGG as a reference resource for gene and protein annotation. *Nucleic Acids Res* **44**, D457-462 (2016).

10 Gillespie, M. *et al.* The reactome pathway knowledgebase 2022. *Nucleic Acids Res* **50**, D687-D692 (2022).

11 Edgar, R., Domrachev, M. & Lash, A. E. Gene Expression Omnibus: NCBI gene expression and hybridization array data repository. *Nucleic Acids Res* **30**, 207-210 (2002).

12 Delorey, T. M. *et al.* COVID-19 tissue atlases reveal SARS-CoV-2 pathology and cellular targets. *Nature* **595**, 107-113 (2021).

13 Tucker, N. R. *et al.* Transcriptional and Cellular Diversity of the Human Heart. *Circulation* **142**, 466-482 (2020).

14 Litvinukova, M. *et al.* Cells of the adult human heart. *Nature* **588**, 466-472 (2020).

15 Trapnell, C. *et al.* The dynamics and regulators of cell fate decisions are revealed by pseudotemporal ordering of single cells. *Nat Biotechnol* **32**, 381-386 (2014).

16 Qiu, X. *et al.* Reversed graph embedding resolves complex single-cell trajectories. *Nat Methods* **14**, 979-982 (2017).

17 Cao, J. *et al.* The single-cell transcriptional landscape of mammalian organogenesis. *Nature* **566**, 496-502 (2019).

18 Liao, Y., Wang, J., Jaehnig, E. J., Shi, Z. & Zhang, B. WebGestalt 2019: gene set analysis toolkit with revamped UIs and APIs. *Nucleic Acids Res* **47**, W199-W205 (2019).

19 Duan, Q. *et al.* L1000CDS(2): LINCS L1000 characteristic direction signatures search engine. *NPJ Syst Biol Appl* **2**, 16015- (2016).

20 Wang, Z., Lachmann, A., Keenan, A. B. & Ma'ayan, A. L1000FWD: fireworks visualization of drug-induced transcriptomic signatures. *Bioinformatics* **34**, 2150-2152 (2018).

**
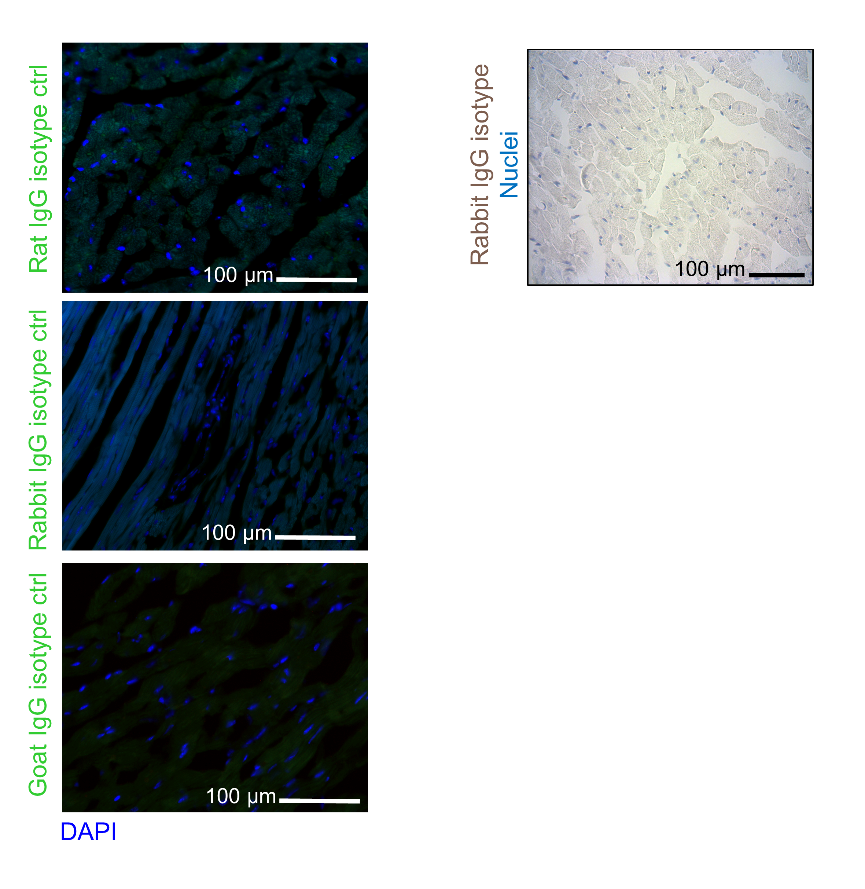
**

Figure. S1.

**Controls for immunostainings of the mouse hearts.** Images show control stainings omitting primary antibodies and including isotype controls and secondary antibodies.


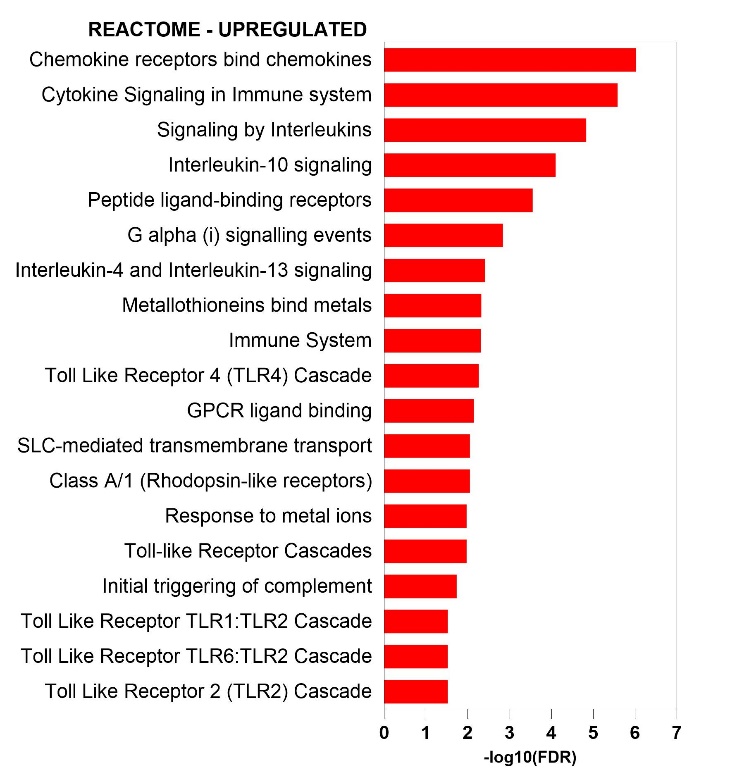

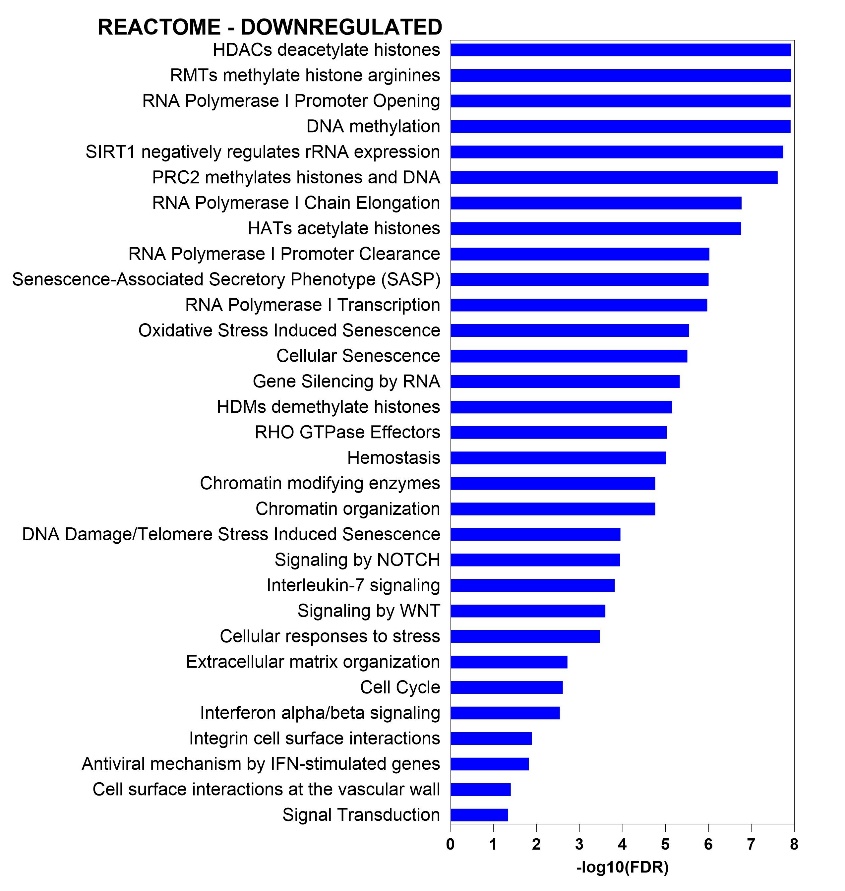
Figure. S2.

**Transcriptomic analysis in human cardiac pericytes challenged with the S-protein.** Bar graphs report the most relevant Reactome pathways differentially expressed in pericytes treated with the S-protein vs. PBS-vehicle control. FDR = false discovery rate. Genes were considered differentially expressed for FDR ≤ 0.1.


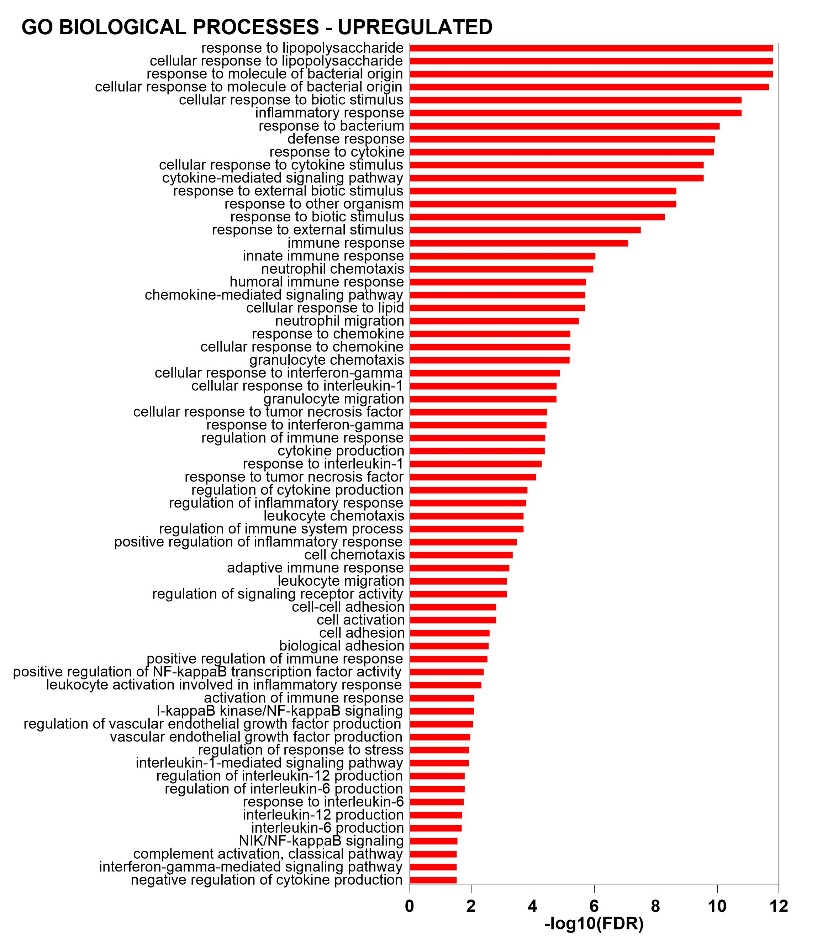
Figure. S3.

**Transcriptomic analysis in human cardiac pericytes challenged with the Spike protein.** Bar graphs report the most relevant GO biological pathways differentially expressed in pericytes treated with the S-protein vs. PBS-vehicle control. FDR = false discovery rate. Genes were considered differentially expressed for FDR ≤ 0.1.


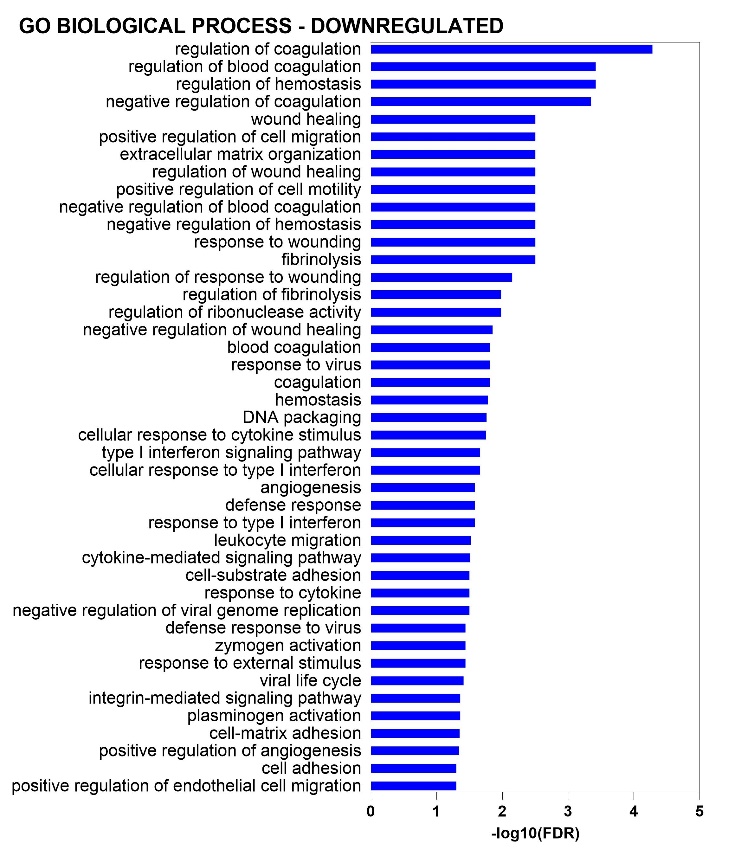


Figure. S3 (continuation).

**Transcriptomic analysis in human cardiac pericytes challenged with the Spike protein.** Bar graphs report the most relevant GO biological pathways differentially expressed in pericytes treated with the S-protein vs. PBS-vehicle control. FDR = false discovery rate. Genes were considered differentially expressed for FDR ≤ 0.1.

| **Cell source** | **Congenital heart defect** | **Patient age** |
| --- | --- | --- |
| Right ventricle | Ventricular septal defect | 6 months |
| Right ventricle | Tetralogy of Fallot | 6 months |
| Right ventricle | Atrial-ventricular canal | 3 years |

Table S1.

Cardiac pericyte donors used for bulk RNA-Sequencing.

|  | | | | | | |
| --- | --- | --- | --- | --- | --- | --- |
| ANTIGEN | **HOST** | **SUPPLIER - CATALOGUE - (CLONE)** | **DILUTION** | **DEVELOPMENT SYSTEM** | **PRE-TREATMENT** |  |
| CD45 | Rat | NovusBio  NB100-77417 (30-F11) | 1:500 | Fluorescence | Citrate buffer, pH 6, 98 ͦ C, 10 min |  |
| Ly6G/6C | Rat | R&D MAB1037 (RB6-8C5) | 1:100 | Fluorescence | Citrate buffer, pH 6, 98 ͦ C, 10 min |  |
| F4/80 | Rabbit | ProteinTech 28463-1-AP | 1:500 | Fluorescence | Citrate buffer, pH 6, 98 ͦ C, 10 min |  |
| PDGFRβ | Goat | R&D AF1042 | 1:50 | Fluorescence | Acetone, -20 ͦ C, 5 min |  |
| IB4 | - | I21414 Life Tech Biotin-conjugated | 1:200 | Fluorescence | Acetone, -20 ͦ C, 5 min |  |
| α-Sarc Actin | Mouse | Sigma A2172 (5C5) | 1:100 | Fluorescence | Acetone, -20 ͦ C, 5 min, or  Citrate buffer, pH 6, 98 ͦ C, 10 min |  |
| C5a | Rabbit | Invitrogen PA578891 | 1:1000 | DAB | Citrate buffer, pH 6, 98 ͦ C, 10 min |  |
| ICAM-1 | Goat | R&D AF796 | 1:40 | Fluorescence | Citrate buffer, pH 6, 98 ͦ C, 10 min |  |

Table S2.

Antibodies used for immunohistochemistry analyses of mice hearts.

Dataset S1. (separate file)

A table summarising results of bulk RNA-Seq analysis of cardiac pericytes challenged with the Spike protein or vehicle *in vitro*. List of KEGG, Reactome, GO Biological Process, and GO Cell Component pathways significantly UP- or DOWN- regulated by the Spike protein.

Dataset S2. (separate file)

A table summarising over-represented gene ontology (biological processes) of coregulated modules of differentially expressed genes across trajectory with significant enrichment of differentially expressed genes from bulk RNA-seq comparing vehicle and spike. The analysis was performed using WebGestalt R package. A gene ontology term was considered to be significant if FDR < 0.05.

WebGestalt (WEB-based Gene SeT AnaLysis Toolkit, http://www.webgestalt.org/) and paper reference: Liao Y, Wang J, Jaehnig EJ, Shi Z, Zhang B. WebGestalt 2019: gene set analysis toolkit with revamped UIs and APIs. Nucleic Acids Res 2019;47(W1):W199-W205. DOI: 10.1093/nar/gkz401ed to be significant if FDR < 0.05

Dataset S3. (separate file)

A table summarising result from L1000CDS2. The table lists the top 50 drugs that have the potential to reverse gene signature after exposure to S-protein. The table is sorted by score, which is the percentage of gene signature overlapping with the genes perturbed after drug exposure. The table also lists the cell line and drug concentration employed for perturbation. We have provided a brief note to signify their potential role.

L1000CDS2 and paper reference: Duan Q, Reid SP, Clark NR, Wang Z, Fernandez NF, Rouillard AD, Readhead B, Tritsch SR, Hodos R, Hafner M, Niepel M, Sorger PK, Dudley JT, Bavari S, Panchal RG, Ma'ayan A. L1000CDS2: LINCS L1000 characteristic direction signatures search engine. NPJ Syst Biol Appl. 2016;2:16015–. doi: 10.1038/npjsba.2016.15. Epub 2016 Aug 4. PMID: 28413689.

Dataset S4. (separate file)

A table summarising over-represented drug target enrichment using DrugBank database. The analysis was performed using WebGestalt. A drug was considered to be significant if FDR < 0.05. WebGestalt (WEB-based Gene SeT AnaLysis Toolkit, http://www.webgestalt.org/) and paper reference: Liao Y, Wang J, Jaehnig EJ, Shi Z, Zhang B. WebGestalt 2019: gene set analysis toolkit with revamped UIs and APIs. Nucleic Acids Res 2019;47(W1):W199-W205. DOI: 10.1093/nar/gkz401ed.
